# The regulation of hyphae growth in *Candida albicans*

**DOI:** 10.1080/21505594.2020.1748930

**Published:** 2020-04-10

**Authors:** Hui Chen, Xuedong Zhou, Biao Ren, Lei Cheng

**Affiliations:** a State Key Laboratory of Oral Diseases, Sichuan University, Chengdu, China; b Department of Operative Dentistry and Endodontics, West China Hospital of Stomatology, Sichuan University, Chengdu, China

**Keywords:** Oral candidiasis, *Candida albicans*, hyphae, mycelium, fungi

## Abstract

In the last decades, *Candida albicans* has served as the leading causal agent of life-threatening invasive infections with mortality rates approaching 40% despite treatment. *Candida albicans (C. albicans)* exists in three biological phases: yeast, pseudohyphae, and hyphae. Hyphae, which represent an important phase in the disease process, can cause tissue damage by invading mucosal epithelial cells then leading to blood infection. In this review, we summarized recent results from different ﬁelds of fungal cell biology that are instrumental in understanding hyphal growth. This includes research on the differences among *C. albicans* phases; the regulatory mechanism of hyphal growth, extension, and maintaining cutting-edge polarity; cross regulations of hyphal development and the virulence factors that cause serious infection. With a better understanding of the mechanism on mycelium formation, this review provides a theoretical basis for the identification of targets in candidiasis treatment. It also gives some reference to the study of antifungal drugs.

With the increasing number of immunocompromised patients [1], the extensive development of organ transplantation, and the wide use of immunosuppressants and antibiotics in cancer chemotherapy [2], the clinical *Candida* infection rate increases yearly [3–7]. The detection rate of *Candida albicans* (*C. albicans*) was 70%~90% among all candidiasis-causing fungi [8–10]. *C. albicans* also accounts for a high proportion of candidiasis patients, and the mortality rate of these patients is up to 43.6% due to candidemia [11].

One important characteristic of *C. albicans* is that it can exist in three phases, budding yeast, pseudohyphae, and hyphae [6]. The plasticity of the mycelial form is a determinant factor of drug resistance and is also an important form during the infection stage [7]. In addition, the transformation of *C. albicans* from yeast to hypha can help fungi escape the phagocytosis of macrophages, resulting in an increased likelihood of invading host tissues and causing greater damage [12]. Therefore, we summarized recent studies from different ﬁelds of fungal cell biology which are instrumental for understanding hyphal growth. This includes research on the differences among *C. albicans* phases; the regulatory mechanism of hyphal growth, extension, and maintaining cutting-edge polarity and the virulence factors that cause serious infection. With a better understanding of the mechanism on mycelium formation, this review provides a theoretical basis for the identification of targets in candidiasis treatment. It also gives some reference to the study of antifungal drugs.

## The differences among yeast, hyphae, and pseudohyphae

*C. albicans* grows and forms mycelia in changing environments in the host, adapting to a variety of micro-ecological environments. Yeast, hyphae, and pseudohyphae differ in their cell morphology, function, and growth conditions [13–17].

Yeast cells, the default cell morphology under most in vitro conditions, are round or oval, have a unicellular morphology, can be involved in biofilm formation, can be toxic or remain symbiotic in blood, and maintain symbiosis in the oral cavity, skin, and vagina [18–21].

**Figure 1.**
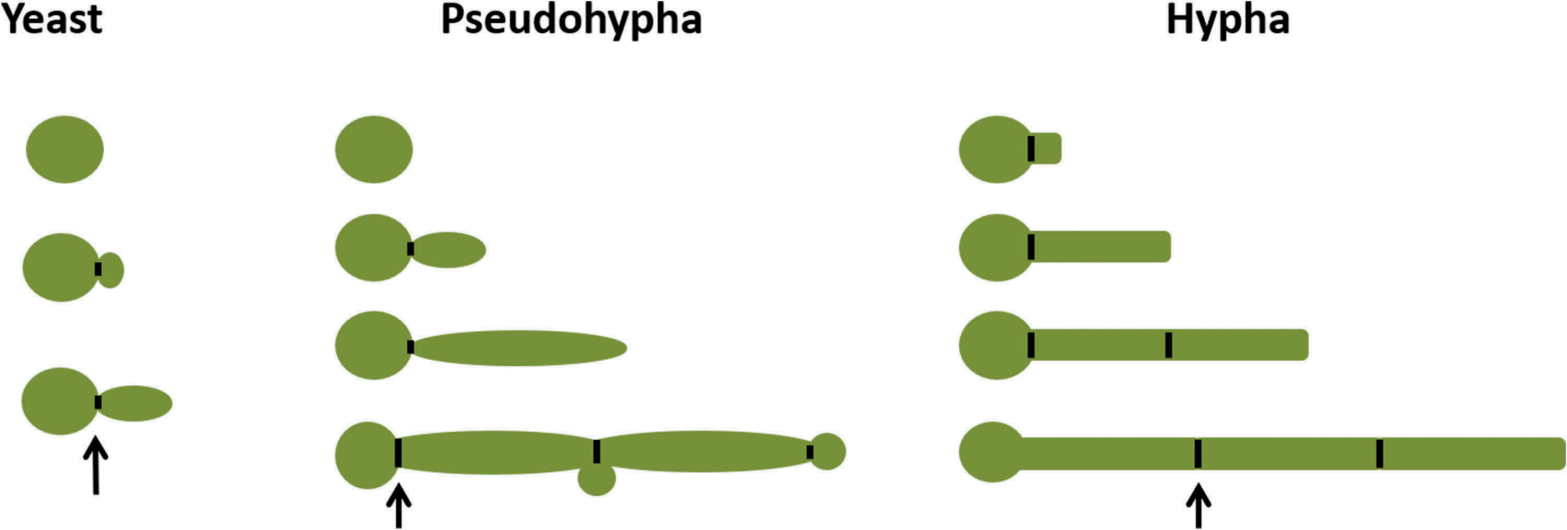
Schematic diagram of yeast, pseudohypha, and hypha (black arrow indicates septin ring).

Pseudohyphal cells have long elliptic, multicellular forms, which can be induced at pH 6.0, 35°C, on solid limited nitrogen medium, and via involvement in biofilm formation [22]. In Pseudohyphal cells, there is a constriction at the neck of the bud and mother cell, even at every subsequent septal junction [23]. Pseudohyphae cells can vary widely in width and length so that at one extreme they resemble hyphae, and at the other, they resemble elongated buds of yeast cells. One of the characteristics of pseudohyphae is that the width of each segment that form the mycelia is not constant, being wider at the center than the two ends (Figure 1). In pseudohyphae, the connection between a mother cell and daughter cell is easily interrupted by mechanical agitation, and this connection is not difficult to interrupt.

Hyphae cells have tubular, multicellular forms, which can be induced by a temperature of 37°C, N-acetyl glucosamine [24–27], embedding matrix [28–30], hypoxia and hypercapnia [31,32], alkaline pH in vitro [33,34], involvement in biofilm formation, and the ability to grow thigmotropically [35]. Hyphae developed from an ungerminated yeast cell, without constriction in the neck of the mother cell and having parallel sides throughout its length. During the hyphal cell cycle, a septin ring will appear in the daughter cell. A simple way to distinguish between hyphae and pseudohyphae is to measure the width of the mycelium, the width of pseudohyphae cells is always larger than the hyphal cells. The width of hyphal cells is about ~2.0 µm on most media. Pseudohyphal cells have a minimum width of 2.8 µm.

## Regulation of hyphal morphology

### Signaling pathways of hyphal morphogenesis

#### Cek MAPK (mitogen-activated protein kinase) pathway

**Figure 2.**
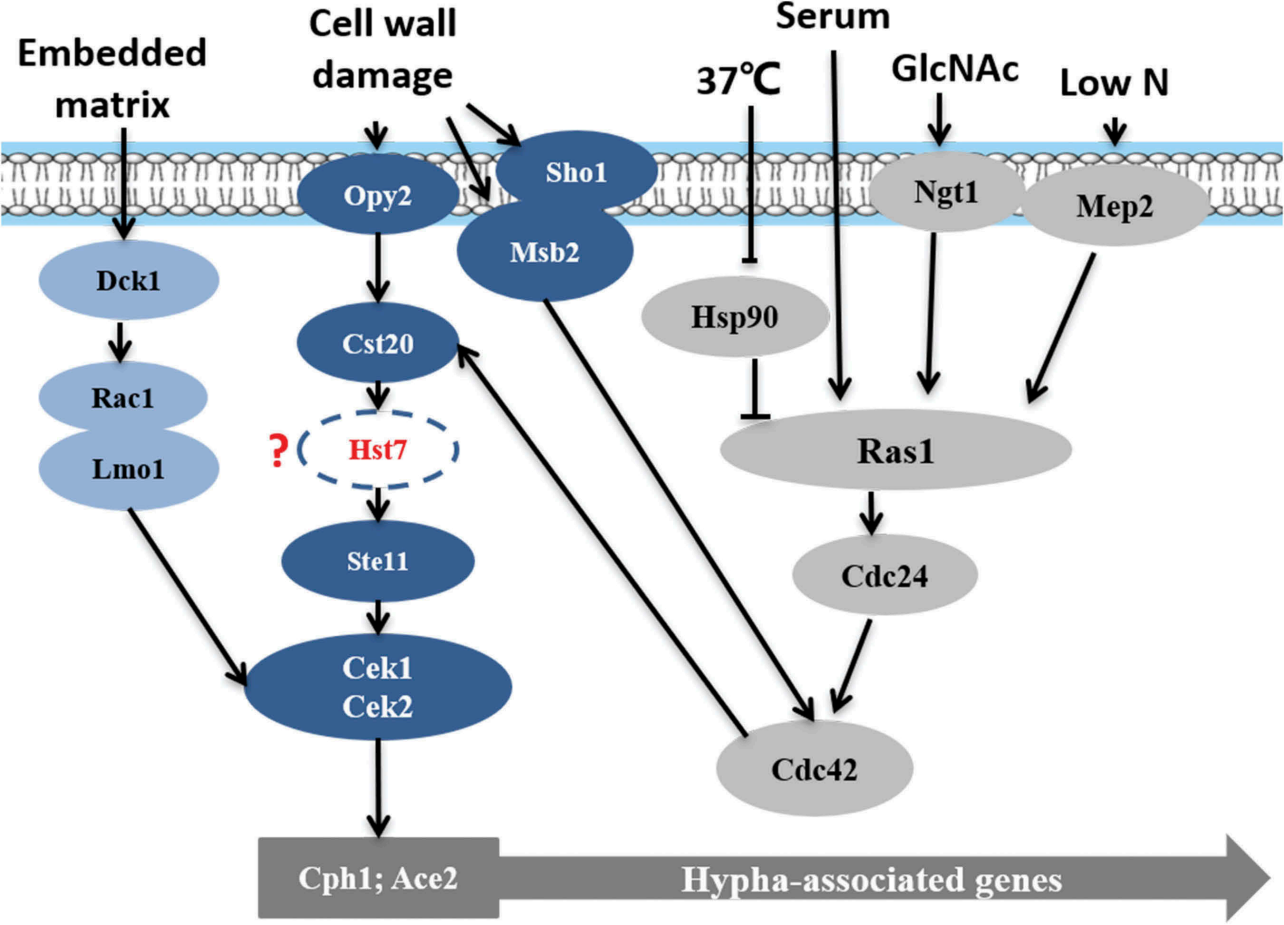
Cek MAPK pathway. The Cek1 mitogen-activated protein kinase pathway (MAPK pathway, dark blue) is induced by the embedded matrix environment (light blue), cell wall damage (dark blue), and low nitrogen (gray) and eventually leads to mycelium formation via the phosphorylation of transcription factor Cph1, Ace2.

Chen et al. first studied the presence of MAPK in *C. albicans* [36,37]. The MAPK pathway is induced by factors such as the embedding matrix environment [37], cell wall damage [38], low nitrogen [39]. Figure 2 is a schematic diagram of the Cek MAPK pathway in *C. albicans*. From the previous study, Opy2, Sho1, Msb2 may form a protein complex that interacts with Cdc42 and Cst20, thus triggering Cek1 phosphorylation [40]. In addition, under osmotic stress, the complex may need to recruit all elements required for polarization/depolarization of cytoskeleton and other related structures such as septin ring [40]. Cells defective in Opy2, Sho1, Msb2 showed lethal phenotypes under osmotic stress, which was presented multinucleated, rounded and abnormally large cells; disorder of actin and myosin cytoskeleton; the septin ring not correctly positioned; and chitin deposition defects. In the previous study, mutant strains formed by deleting *CST20, HST7, CEKl,* or *CPH1* and grown on Spider solid culture medium could not form hyphae [32,41,42]. However, the high expression of *CPH1* can mitigate the loss of *CEK1, CST20* [43–45]. Recent studies suggested that Hst7 may lie between Cst20 and Cek1/Cek2 [37]. Moreover, *CPH1* overexpression can induce the expression of *CEK1, CEK2*, and genes that are similar to the pheromones of *Saccharomyces Cerevisiae*, indicating that they may be regulated by the MAPK pathway [46–49]. Ace2, a transcription factor, has multiple functions in cell separation, hyphal morphogenesis, and biofilm formation and works with Efg1, Brg1, and Bcr1 to regulate transcription [50].

#### cAMP-PKA (cyclic adenosine monophosphate—protein kinase A) pathway

**Figure 3.**
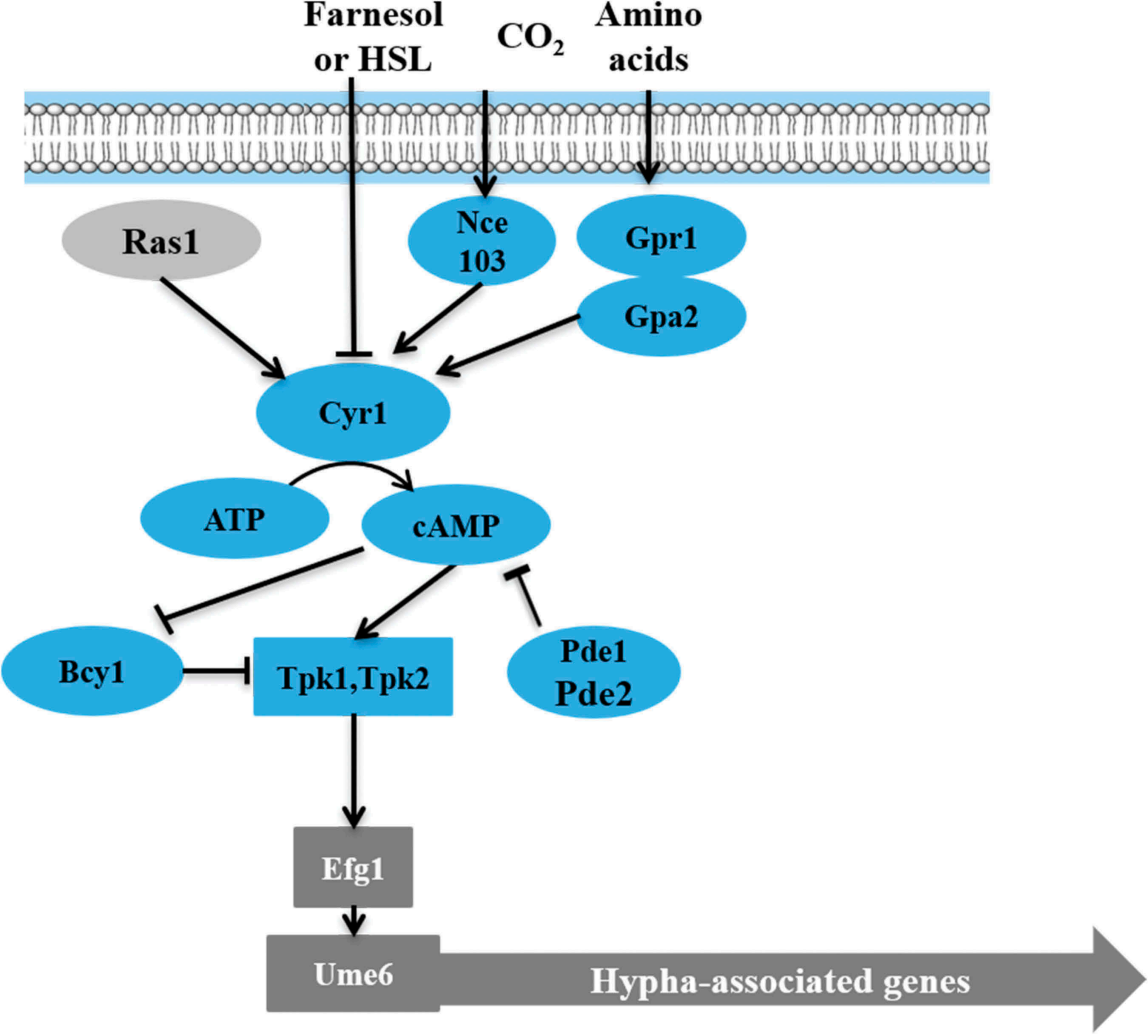
Regulatory models of cAMP-PKA signaling pathway in *C. albicans.*

The cAMP-PKA pathway plays a key role in *C. albicans* growth, morphogenesis, glycogen synthesis, mitochondrial activity, and energy metabolism [51–53]. cAMP is necessary for the activation of PKA pathway in response to various environmental stimuli, including serum, N-Acetylglucosamine (GlcNAc), amino acids, and carbon dioxide. Intracellular cAMP levels are regulated by phosphodiesterase (Pde2) and adenylyl cyclase (Cyr1) [54–56] (Figure 3). In the mouse model of systemic infection, loss of *PDE2* resulted in reduced both hyphal growth and virulence [57].

In *C. albicans*, Tpk1 and Tpk2 have distinct roles in hyphal development under different filamentous induction conditions [58–61]. On solid medium, Tpk1 is involved in filamentation, while in the liquid medium, Tpk2 is involved in hyphal growth [51,59,61,62]. Interestingly, the *C. albicans tpk1/tpk1, tpk2/tpk2* double deletion mutants showed no hyphal growth under various test conditions [63]. However, *bcy1Δ/Δ* was acquired in a *tpk2Δ/Δ* background, which reduced PKA activity, with two distinct phenotypes: smooth and filamentous [64,65]. Smooth *bcy1Δ/Δ* cells showed defects in hyphal growth, while filamentous *bcy1Δ/Δ* exhibited hyphal growth indistinguishable from the wild type [65]. Furthermore, the cAMP-PKA pathway may be a promising target for antifungal drug development since it is conserved in eukaryotic cells and regulates virulence in *C. albicans.*

#### pH-response pathway

Phenotypic plasticity is one of the most prominent characteristics of *C. albicans*. Different cell forms play different roles in infection and adaptation to different host niches. Acidic pH values inhibit the transformation of yeast into filamentous cells, while neutral and alkaline pH values promote the formation of filamentous cells [66,67]. *C. albicans* can also actively regulate the environmental pH through the metabolism of effective nutrients.

**Figure 4.**
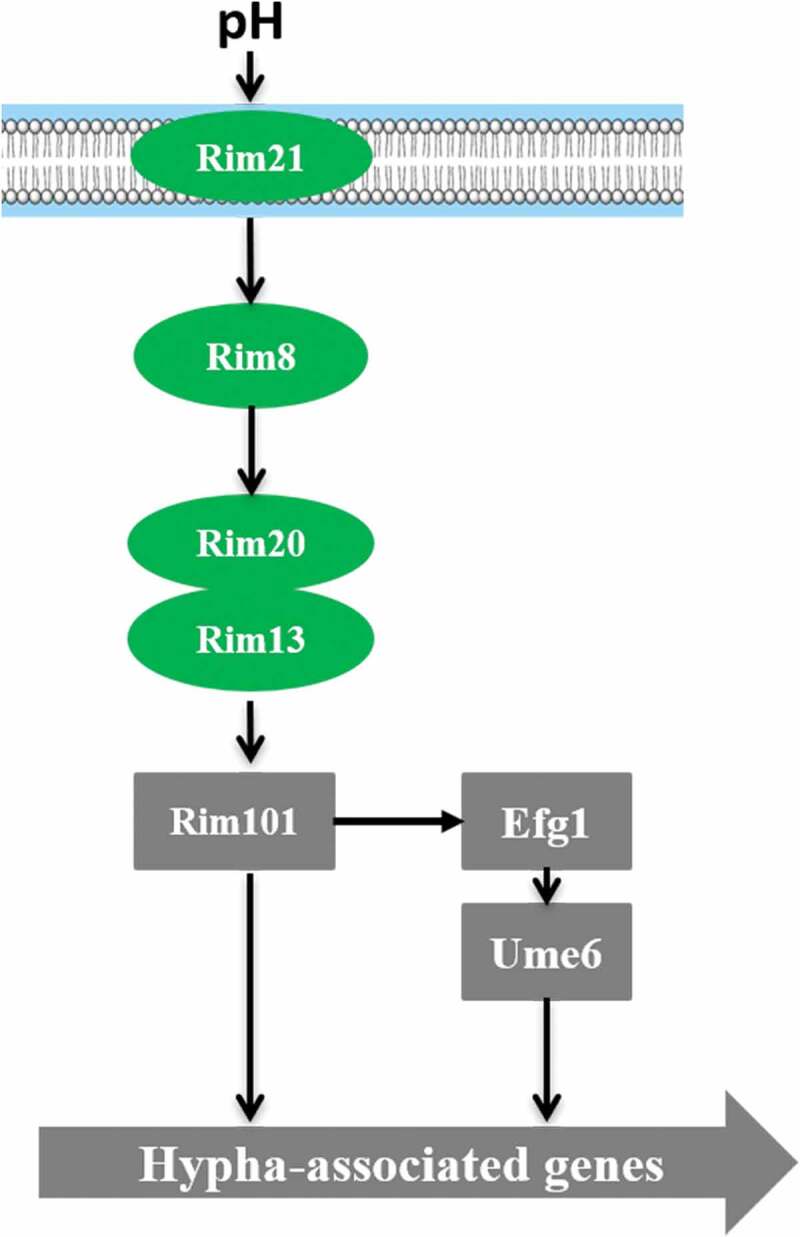
The Rim101‑pH sensing pathway.

Figure 4 shows a schematic representation of the Rim101‑pH sensing pathway in *C. albicans*. Rim101 regulates pH-controlled morphologic transition and virulence. Deletion of *RIM101* inhibits alkaline pH-induced filamentation [68]. *PHR1* and *PHR2* are two genes encoding beta-1,3- and beta-1, 6-glucan crosslinking glycosidases in *C. albicans*, which are regulated differently by Rim101 under different pH conditions [68]. *PHR1* is expressed at pH >5.5, while *PHR2* is expressed at pH<5.5. The *phr1Δ/Δ* mutant exhibits growth defects in alkaline pH conditions, while the *phr2Δ/Δ* mutant grows poorly in acidic pH conditions. Rim101 has been shown to be processed into different versions of the protein under acidic and alkaline conditions [69]. Rim101 is processed to a 74-kD active form at alkaline pH, while to a 65-kD protein with unknown function at acidic condition [69]. The Rim101 activation process also requires three other proteins, Rim8, Rim20, and Rim13 [70,71] (Figure 4). Studies show that the absence of any one of these four types of proteins can lead to loss of the ability to form hyphae and affect the virulence and adhesion to epithelial cells [71–74].

#### Hog MAPK pathway

**Figure 5.**
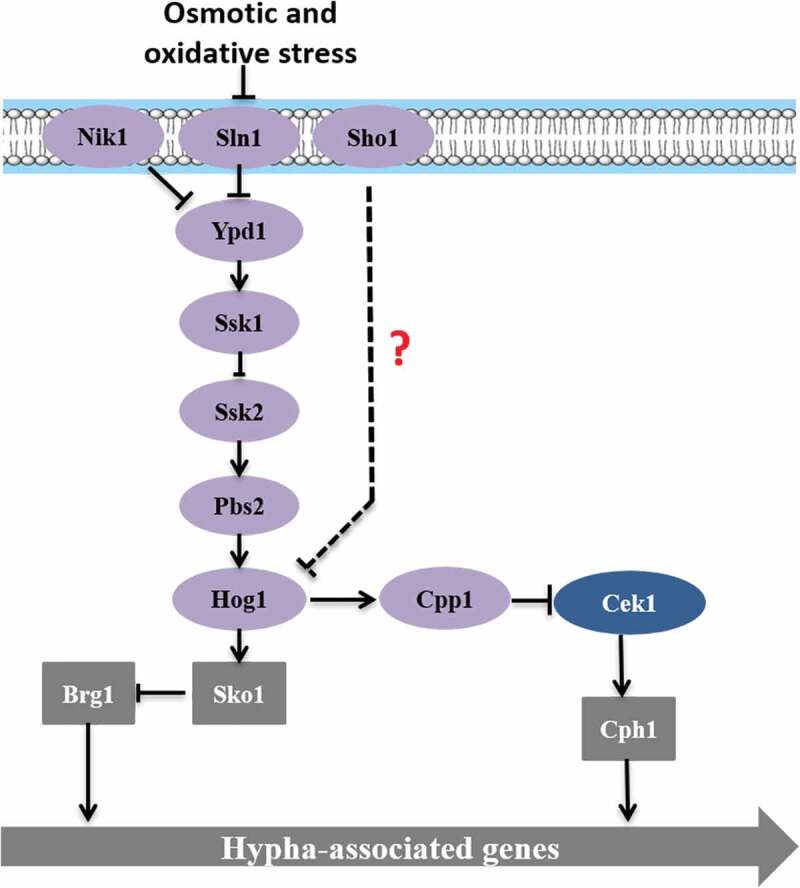
Hog MAPK pathway.

The Hog MAPK pathway (Figure 5), which is a histidine-kinase pathway that was first found in bacteria, primarily regulates the bacterial resistance and the degradation of aromatic compounds. In *C. albicans*, the Hog MAPK pathway can regulate mycelium formation, which affects the virulence of the strain, and may even participate in regulating the oxidative stress response [38,75]. *C. albicans* accumulates glycerol by partially Hog1-dependent pathway under osmotic stress [76]. Hog1 is phosphorylated by hyperosmotic shock and then transferred to the nucleus [77]. Hog1 inhibits the yeast to hyphae transition by downstream elements. This repression is independent of Efg1 and Cph1 since a triple mutant of *hog1, efg1* and *cph1* shows the hyperfilamentous phenotype characteristic of *hog1* single mutant [78]. Deng and Lin found that either *HOG1* or a phosphatase gene *CPP1* null mutant from both white and opaque cells represented a hyperfilamentous morphology, and *HOG1* and *CPP1* genes are involved in regulating Cek1 phosphorylation, indicating that the Cpp1 phosphatase may act as a key intermediary between the Hog1 and Cek1 in *C. albicans* [79]. The Hog pathway is also involved in cell wall biogenesis [78]. In addition, the two-component phosphorelay system Sln1-Ypd1-Ssk1 has also been reported to activate Hog1 under different stress. Sln1, a membrane-bound sensing protein, which is autophosphorylated on a histidine residue. The Sho1 branch is formed by Sho1 transmembrane protein. However, the role of Sln1and Sho1 as upstream components of the Hog pathway is still uncertain, since no clear phenotype has been observed either alone or in combination with other histidine kinase analyses [80]. Studies have shown that the *SSK1* deletion significantly increases the risk of *Candida* cell killing by multicore neutrophils [32,45]. The absence of any protein in the pathway does not affect the cellular response to osmotic pressure, but it can weaken the mycelium formation in Spider culture medium and the toxicity in a mouse system infection model [45,81].

#### Tup1-mediated negative regulatory pathway

**Figure 6.**
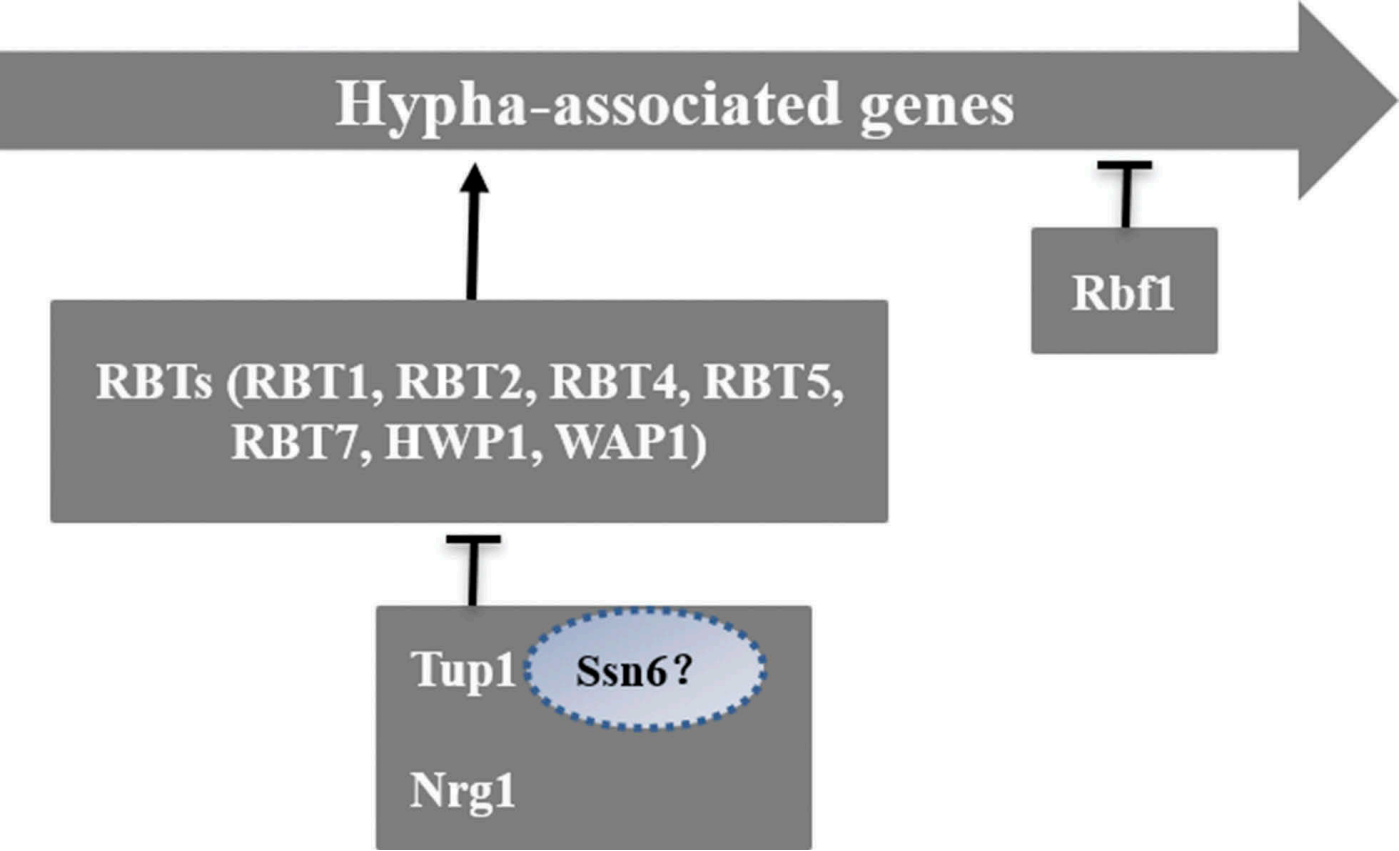
Tup1-mediated negative regulatory pathway.

The negative regulation of mycelium formation is primarily mediated by the universal transcription factor Tup1 in synergism with Nrg1 or mycelial growth regulation factor Rfg1 [82] (Figure 6). Tup1, a sequence-specific DNA-binding protein, can repress transcription, and Tupl-deficient strains can form hyphae effectively without any specific induction conditions [83,84]. RBTs repressed by Tup1 include *RBT1, RBT2, RBT4, RBT5, RBT7, HWP1,* and *WAP1*. Rfg1 is a DNA-binding protein that acts downstream of the Tup1 pathway [85]. The absence of Rfg1 can promote the formation of mycelia and the expression of some mycelium-specific RBT genes [83,86]. Nrgl is a zinc finger DNA-binding protein, and its absence can also promote mycelium formation and the expression of some RBTs. Recent studies have shown that the Nrg1 mRNA level in *C. albicans* cells decreases at 37°C, thus weakening the inhibition of hyphae formation [84]. A DNA microarray analysis found that there are 61 genes whose expression rises significantly during the process of hypha formation when cells are exposed to 37°C and serum factor [86]. Approximately half of these genes are inhibited by Tup1, Nrg1, and Rfg1, indicating that this pathway plays a very important negative regulatory role in mycelium formation [82,84,85]. Cells that lack these repressors will grow to become pseudohyphae, and the expression of specific mycelium genes can be suppressed [83,86]. The prevailing view is that Tup1 and Ssn6 were required for Nrg1-mediated inhibition in *C. albicans* [87]. However, whether Ssn6 can independently regulate hypha-specific genes under certain conditions remains to be proved.

### Regulation of hypha elongation

**Figure 7.**
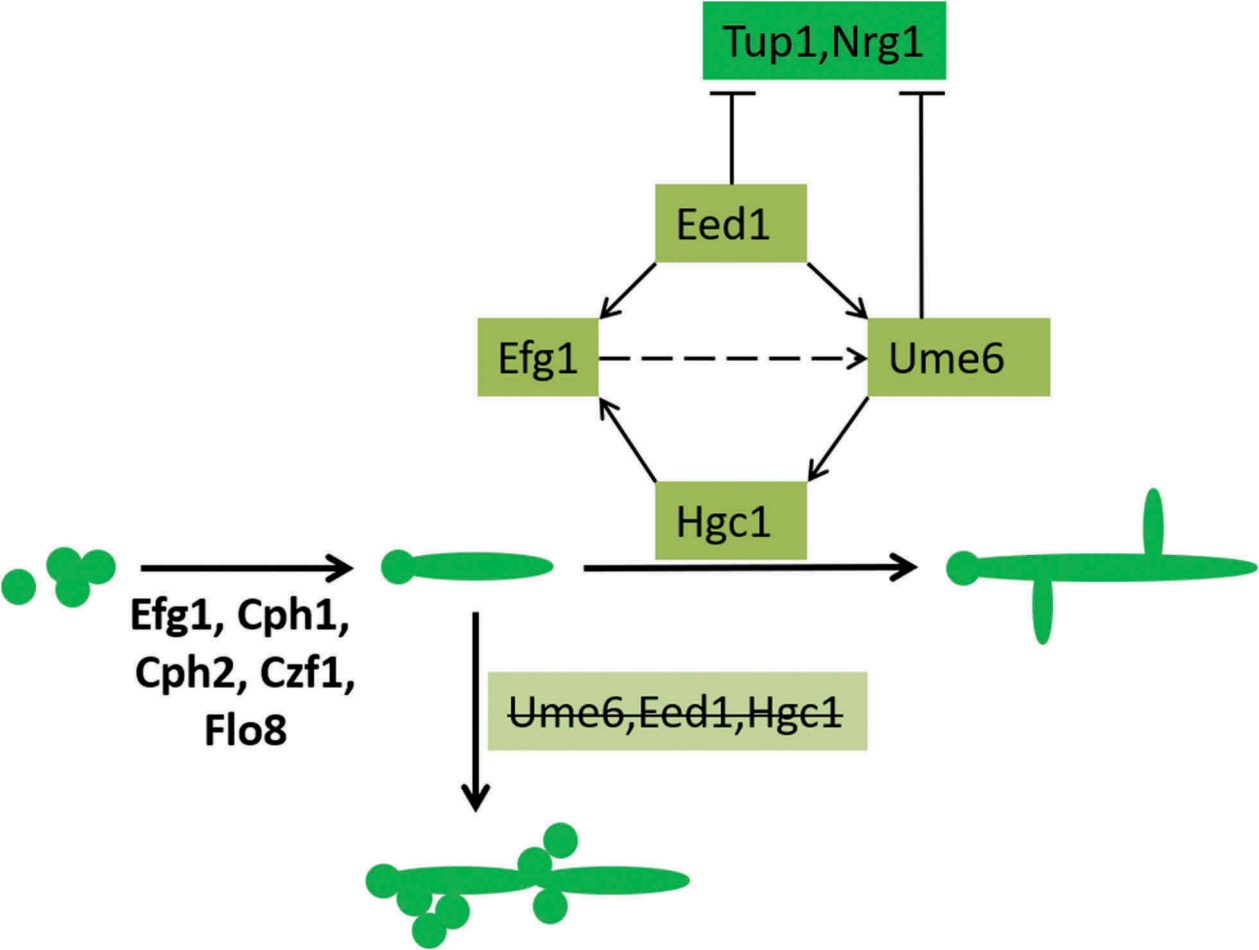
Key factors of mycelial elongation. Transcription factors such as Efg1 and Cph1 are involved in this regulation process. Eed1, Hgc1, Ume6 play key roles in mycelial elongation. Ume6 and Eed1 also negatively regulate Tup1 and Nrg1.

Ume6, Eed1, and Hgc1 play important roles in hypha elongation, as shown in Figure 7 [88–90]. The transcription factors Efg1, Cph1, Cph2, Czf1, and Flo8 are also involved in this elongation process. Eed1 is required for *UME6* expression and plays an important role in mycelial maintenance [90]. In an *efg1-/-* mutant strain, *EED1* overexpression will partially compensate for mycelium formation. Ume6 can compensate for the phenotype of an *eed1-/-* mutant strain. Therefore, Eed1 is located between Efg1 and Ume6 in the pathway [90]. Ume6 and Eed1 can also negatively regulate Tup1 and Nrg1 [91] and indirectly promote the formation of mycelia. Studies have shown that tube formation in *eed1-/-* and *ume6-/-* strains in liquid culture medium at the first stage simply remain in the induction stage, and the cells cannot continue to grow [91]. On a solid Spider culture medium, these mutant strains can only form smooth colonies without mycelia [91]. Hgc1 is the periodic protein partner of Cdc28 (also known as Cdk1) in the cell cycle. Hgc1 has multiple roles in cell polarity growth, which inhibits mycelial cell secretion. *hgc1-/-* mutant strain cells can only form very short bud tubes [92]. In a *ume6-/-* mutant strain, *HGC1* expression can be induced but cannot be maintained. Therefore, the maintenance of *HGC1* expression depends on Ume6 [88,93].

### Mechanism of mycelial polar growth

**Figure 8.**
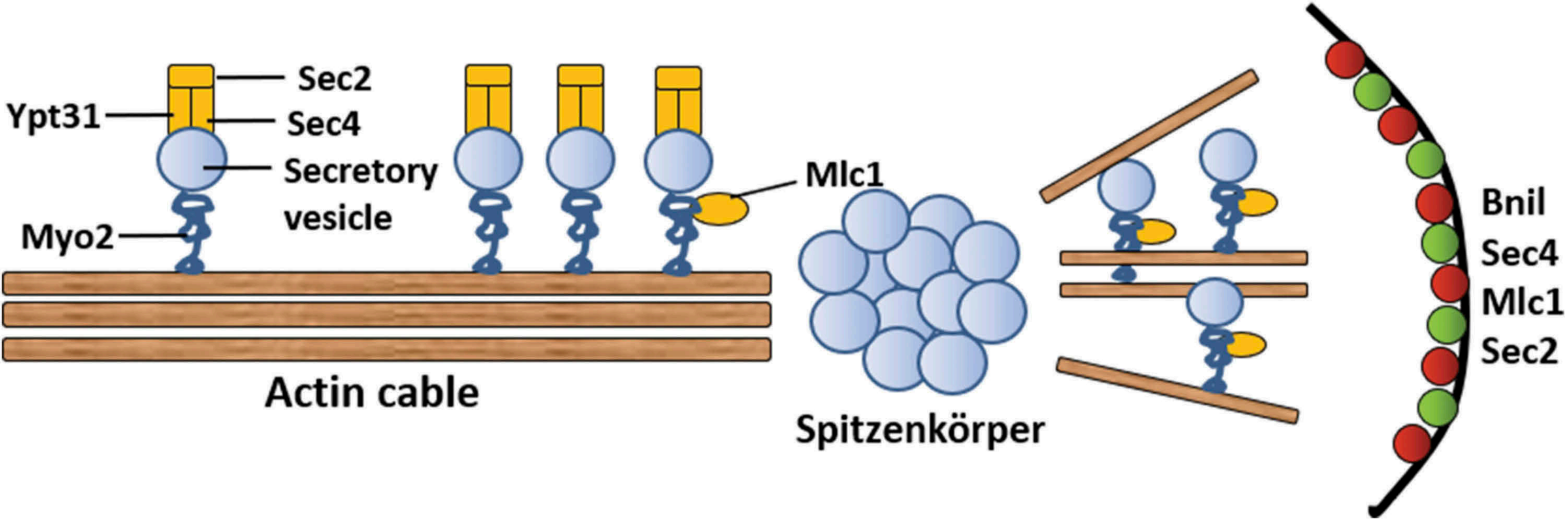
Transport secretory vesicles to mycelium tip. The secretory vesicles are transported to the top by actin. All vesicles will carry Sec4. After forming Spitzenkörper, the vesicles are polarized by the polar bodies and transported to the cell surface by a motorized protein strip.

When mycelium elongation, the tip needs to maintain polar growth, which requires the continuous transport of vesicle secretion factors of the membrane envelope to the polarity growth site. These vesicles provide materials to elongate the membrane and enzymes to synthesize new cell walls. The specific process is as follows (Figure 8): the Golgi body secretes vesicles, Ypt31 on the vesicle surface activates Sec2, and Sec2 activates Sec4 to form a complex. This complex can combine with myosin Myo2 and actin filaments and then be transport to the tip. Myosin Mlc1 can also provide power for transport. Studies have found that blocking the action of actin can cause the hyphal tip to swell because polarity growth is transformed in all directions [94,95]. Deleting the genes for actin Sla1, Sla2, Pan1, Wal1, Vrp1, Myo5 can cause defects in mycelial growth and endocytosis [96–99]. Formin Bni1 and vesicle-related proteins, such as Sec4, Mlc1, and Sec2, aggregate at one point on the tip of the mycelium during three-dimensional rendering. This localization pattern suggests the presence of Spitzenkörper, a structure that was known to drive the tip growth of filaments [100]. The Spitzenkörper is a subterminal region with abundant vesicles that are both exocytic and endocytic in origin. It is thought to be the physical representation of an imaginary vesicle supply center in a model where secretory vesicles radiate from a point source, keeping a certain distance from the end of hyphal. In addition, the mycelial surface has a crescent distribution of exocyst components and polar bodies. The exocyst components help vesicle exit from Spitzenkörper and reach the top surface of the mycelium [100].

## Cross regulations of hyphal development

**Figure 9.**
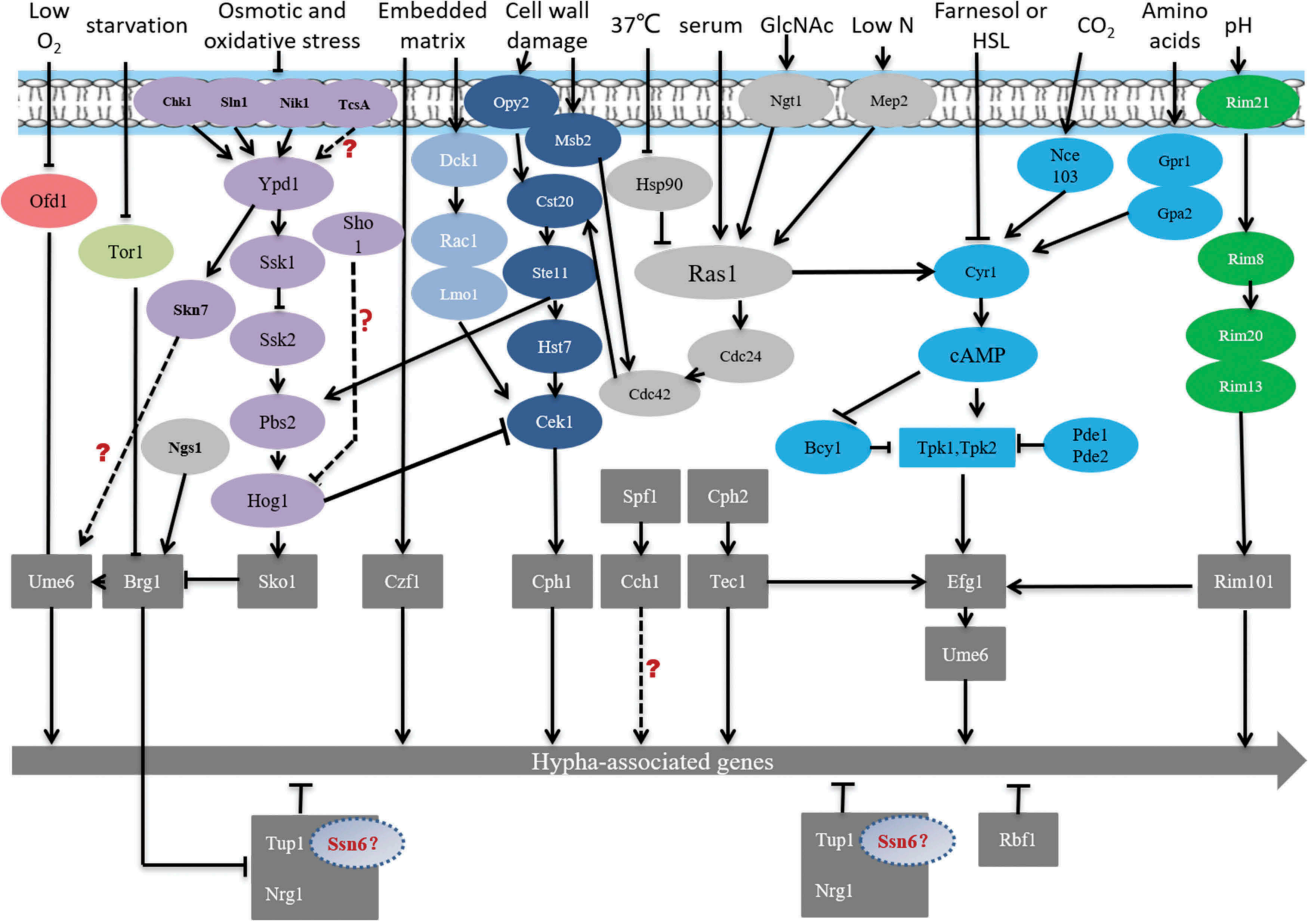
The pathway of hyphal formation. Different colored circles indicate different signaling pathways. The gray rectangles represent transcription factors. “?” represents that the mechanism is still uncleared here.

Except for the classic mycelium formation regulatory pathway mentioned above, there are other cross-regulatory pathways to maintain hyphal development (Figure 9). The previous study found that Spf1, which involved in cell calcium survival pathway, regulates hyphal development and biofilm formation and virulence [101]. Ume6 is a master regulator of filamentation in vitro conditions which null mutants lead to a well-documented filamentation defects. However, Witchley et al. [102] reported ume6 null mutants have the ability to carry out normal morphogenesis in gut by activating the expression of a pro-inflammatory secreted protease, Sap6, and a hyphal cell surface adhesin, Hyr1. The results suggest that Ume6 is not essential for morphogenesis in the gut. At least one fungal filamentous activator must be able to compensate for the loss of Ume6 to regulate gut filamentation. In addition, Su et al. [103,104] found that hyphal induction without inoculation is triggered by Brg1-mediated removal of Nrg1 inhibition. GlcNAc, as well as serum or neutral pH, stimulates the filamentation of log phase cells by down-regulating *NRG1* via transcription [105]. Instead of cAMP-PKA pathway, GlcNAc sensor Ngs1 binds to GlcNAc and activates its N-acetyltransferase activity, leading to *BRG1* expression. The increased *BRG1* level could down-regulate *NRG1* transcription, leading to hyphal growth. Skn7 is a conservative fungal heat shock factor-type transcriptional regulator that is part of a two-component signal transduction system. Previous studies have also revealed a close functional interaction between Skn7 and major morphogenetic regulators (including Efg1, Cph1, and Ume6), suggesting that Skn7 is part of the transcriptional pathway that controls filamentous growth of *C. albicans* [106].

## Virulence factors after mycelium formation

The signal transduction pathway described above is a transcription program of a specific mycelial gene. Increases in gene expression during the process of hypha formation can be identified using gene chip technology [107,108]. Highly expressed genes encode virulence factors such as cell wall-dissolving enzymes and adhesion factors such as secreted aspartic proteases (Saps) and agglutinin-like sequence (Als) that allow *C. albicans* to adhere to, invade, and damage epithelial cells [109]. These factors can enhance the virulence of *Candida albicans*. Although these proteins are associated with hyphal properties, none is necessary for the formation and maintenance of mycelium.

### Invasive enzymes

After mycelium formation, *C. albicans* destroys the host cell membranes by producing Saps. Saps have an extensive substrate specificity of human proteins, such as albumin, hemoglobin, keratin, collagen, laminin, firestone, mucin, and almost all immunoglobulin, including immunoglobulin A, which is resistant to most of the bacterial proteases. There are 10 genes in SAP family (*SAP1–SAP10*) which have a vital role in virulence of *C. albicans* by degrading host tissue proteins as well as adhere to epithelial host tissue. The researchers detected the presence of *SAP1* to *SAP7* genes in all susceptible or drug-resistant *C. albicans* isolates. Previous studies have shown that Sap1~3 has a direct effect on tissue damage during superficial infections, while Sap4~6 seems to be more important for penetration into deeper tissues and interactions with the cellular defense [110]. Jessica N. Witchley [102] has reported that Ume6, a main regulator of filamentation, inhibits gut colonization by activating the expression of *SAP6* and *HYR1*, rather than by effects on cell shape. *SAP6* deletion strains exhibit increased colonization fitness, whereas *SAP6*-overexpression strains exhibit attenuated in the gut. Recently Sap8 has been found to be involved in the cleavage of a signaling glycoprotein, which leads to the activation of the Cek1-MAPK pathway [111]. Previous data provided the evidence of *SAP7-10* expression in *C. albicans* strains under human serum influence and hypothesized that *SAP7* is essential for *C. albicans* survival and helps the cells to escape from the bloodstream. Thus, *SAP7* may help the fungus to cause systemic infections. The expression of *SAP9* in serum was associated with hyphal invasive growth at the site of infection [112]. Previous studies revealed a correlation between prevalence of *SAP9* and *SAP10* genes and the strong biofilm producers by *C. albicans* isolates, as 66.7% of these isolates have both Sap9 and Sap10. Sap 9 and Sap10 enzymes maintain cell surface integrity of the *Candida* cell wall and promote biofilm formation [113]. Recent studies have found that patients infected with fluconazole-resistant *C. albicans* can enhance the Sap production when treated with this drug [110].

### Adhesion

Other major virulence factors expressed after hypha formation are the adhesions, which include agglutinin 1, Als1, mycelium 1, and integrin 1 [114–117]. During the process of infection, they mediated the adhesion of *C. albicans* to the cells, providing conditions for additional invasion [28,118]. The most common type of adhesion is Als. *ALS1* mediates adherence to epithelial cells by encoding a cell surface protein. In *C. albicans*, disruption of both copies of *ALS1* reduces adherence to endothelial cells by 35%, and overexpression of this gene increases adherence by 125%. Recently, some researchers demonstrated that Als3 acts as a type of fungal invasion by binding to the E-, N-calcium adhesive proteins on host cell surface, mediating endocytosis effects, and then invading the host cell [119–121]. Only Als1 and Als5 in Als family have a function similar to that of Als3. *C. albicans* strains lacking *ALS5, ALS6*, or *ALS7* have a common phenotype of increased adhesion and slightly slower growth. Transcription levels of these genes were consistently lower than other ALS genes. Phenotypic results for *ALS5, ALS6,* and *ALS7* suggested that their proteins have redundant functions or even that they have a role in multiprotein complex [122]. Interestingly, previous studies presented here highlight a number of characteristics of these genes and proteins that remain to be explored in *C. albicans* [122]. Als9, Als2, and Als4 have not been tested experimentally [109].

## Summary

This review described the differences among different phases; the growth, extension and cutting-edge polarity of the mycelial regulatory mechanism, and the hyphal virulence factors. The precise regulatory mechanism of mycelium formation was detailed, and a theoretical basis was provided to identify targets for candidiasis treatment.

Although recent advances in fungal cell biology have begun to illuminate the mechanism of hyphal growth, much remains to be understood. One of the remaining issues is the role of Spitzenkörper in the hyphal tip. How Spitzenkörper exactly focusses on the hyphal tip is unclear. Moreover, the interaction between the carrier and the function of motility proteins is not fully understood. Clearly, motor proteins depend on each other to form functional networks, but how their activity is ﬁne-tuned is unclear. Thus, the secretory mechanism of cytoskeletal polarization, which will be meaningful to elucidate the mechanism of apical protein synthesis in further studies. Although gene chip technology has identified many genes, few genes are involved in the polarity of hyphal growth, which requires additional research.
